# Recent Advances on the Functionalities of Polyoxometalate-Based Ionic Liquids

**DOI:** 10.3390/molecules29133216

**Published:** 2024-07-06

**Authors:** Hongxue Wang, Bao Li

**Affiliations:** State Key Laboratory of Supramolecular Structure and Materials, College of Chemistry, Jilin University, Changchun 130012, China; whx23@mails.jlu.edu.cn

**Keywords:** ionic liquid, polyoxometalate, functionality, oxidation reaction, adsorption

## Abstract

Polyoxometalate (POM)-based ionic liquids (POM-ILs) are gaining increasing attention due to their diverse structures and functionalities. POMs in POM-ILs not only act as essential structural building blocks but also play a crucial role in their functional performance. With the incorporation of POMs, POM-ILs find applications in various fields such as chemical catalysis, energy science, materials science, sensors, and more. The abundant availability of POMs and other building blocks in POM-ILs, along with their versatile combination possibilities, present promising opportunities for the future. Rather than focusing solely on discovering new structures of POM-ILs, current developments in this field emphasize exploring their functions, leading to the emergence of numerous new applications. Summarizing these advancements aids in understanding the latest trends and facilitates rapid evolution. This review examines the recent five years’ worth of results to analyze the new functions of POM-ILs, categorizing them based on their unique characteristics.

## 1. Introduction

Ionic liquids (ILs) are a type of salt with a melting point below 100 °C, typically made up of organic cations and inorganic anions. Since Paul Walden successfully synthesized the first IL [1], it has experienced rapid development due to its various advantages, including extensive solubility for organic and inorganic materials, high chemical and thermal stabilities, and low volatility [2,3,4]. These exceptional properties make ILs ideal candidates for applications in catalysis, separation, materials science, and energy science. The diverse properties and broad applications of ILs stem from their varied structures, where anions and cations can be individually tailored to meet specific requirements. According to the compositions, ILs can be roughly divided into two types: one is formed by small molecules, and the other comprises polyelectrolytes or polymers, which both show similar properties [5]. In addition, as the alternatives of ILs, deep eutectic solvents (DESs) are also environmentally friendly liquid media formed by the self-binding of multiple components through supramolecular interactions and can be used in electrocatalysis, oxidative desulfurization [6,7,8,9], etc. In comparison to organic cations, inorganic anions generally play more significant roles in adjusting the functions of ILs, making the introduction of various inorganic components like metal clusters and coordination compounds into ILs a crucial consideration.

Polyoxometalates (POMs) are nano-scale anion clusters formed by the high oxidation state of early-transition metal ions (such as W, Mo, V, etc.) through the point-, edge- and face-sharing O atoms [10]. Depending on the chemical compositions and combination styles, POMs adopt various classical structures, including Keggin [11], Anderson [12], and Wells–Dawson [13], and exhibit diverse properties in medicine [14], catalysis [15], sensors [16], and magnetism [17]. A notable characteristic of POM clusters is their multiple negative charges, making them ideal building blocks for constructing supramolecular assemblies through electrostatic interactions. Additionally, the O atoms on the surface of POM act as strong hydrogen bonding donors, further enhancing the stability of the assemblies. The inherent structural and property features of POM make it suitable as an inorganic anion for binding with organic components like uracil [18], triol ligands [19,20], organic macrocycles [21], etc. Currently, there is a growing body of literature on the preparation of POM-containing ILs (POM-ILs). Compared to other ILs, POM-ILs offer enhanced structural and compositional adjustability due to the incorporation of POMs, along with a variety of functions.

**Figure 1:**
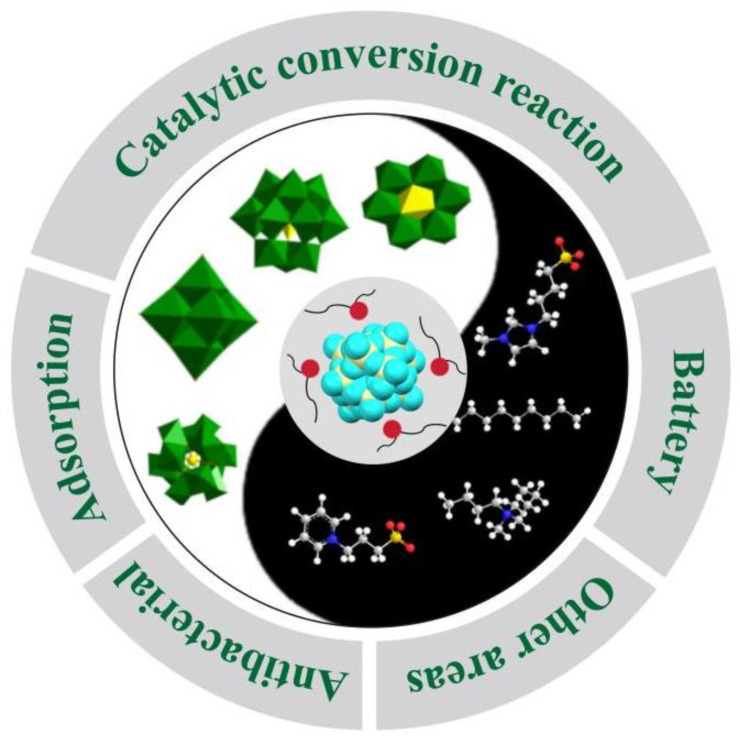
Overview of POM-ILs in this review.

Since the initial study by Emmanuel P. Giannelis [22], POM-ILs have garnered significant attention, with numerous works highlighting their structural and functional advancements. While comprehensive reviews focusing solely on POM-ILs remain scarce, they are briefly mentioned in broader reviews from various perspectives, including phase transfer catalysis [23], self-assembled materials [24], charge balance [25], desulfurization [26,27,28], catalysis [29], stabilization of metal oxoclusters [30], hybrid POMs [31], water oxidation [32], and carbon dioxide conversion [33]. Following the last review dedicated to POM-ILs in 2020 [34], substantial progress has been made in the field of POM-ILs. Given the significance of POM-ILs, it is imperative to summarize the recent advancements in this field over the past five years. Previous discussions have extensively covered the role of POMs in constructing POM-ILs with diverse structures [34], while recent developments primarily focus on the practical applications of POM-ILs. Therefore, this review aims to showcase the novel functions of POM-ILs. It seeks to provide a comprehensive overview of the latest findings in POM-IL applications and identify potential research areas for the future by analyzing current challenges. The review includes an illustrative overview diagram presented in Figure 1.

## 2. The Applications of POM-ILs in the Oxidation Reactions

POMs have been extensively utilized in catalysis for their unique performance advantages, such as adjustable acidity, excellent oxidation properties, high activity, structural stability [35], and robust antioxidant decomposition ability. ILs exhibit properties like thermal stability, chemical stability, and low volatility, making them suitable as an extractant, eco-friendly solvent, or catalyst carrier in catalytic processes. This enhances the stability and catalytic activity of the catalyst, providing distinct advantages in catalysis [36]. Therefore, incorporating POMs into ILs is a green synthesis approach for various substances and a solution to current pollution issues. This section delves into the use of POM-ILs as catalysts in oxidation reactions.

### 2.1. Oxidative Desulfurization

**Figure 2:**
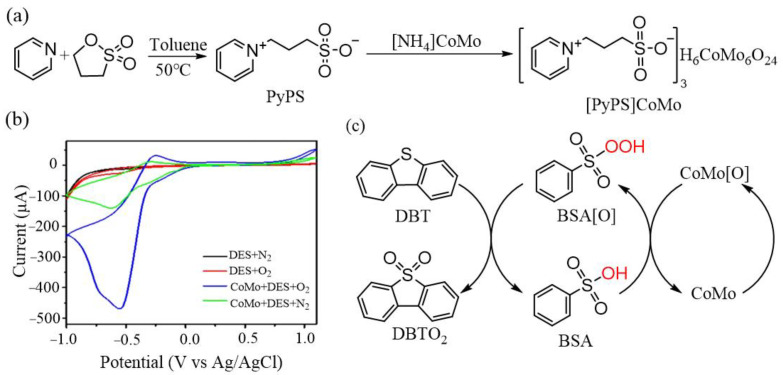
(**a**) Synthetic route of [PyPS]CoMo. (**b**) Cyclic voltammogram curves of various systems, DES in N_2_ or O_2_ or CoMo + DES in N_2_ or O_2_. (**c**) Proposed dual activated catalytic cycle for the biomimetic oxidative desulfurization. Reproduced with permission from [48]. Copyright (2020) Elsevier.

As the demand for energy, especially fuels, rises, the production of sulfur-free clean fuels becomes increasingly urgent. Currently, fuel desulfurization treatment technologies include hydrodesulfurization [37,38], oxidative desulfurization [39,40], biological desulfurization [41,42], extractive desulfurization [43,44] and adsorption desulfurization [45,46]. Among these, oxidative desulfurization is considered the most promising method for obtaining ultra-clean fuels due to its mild reaction conditions, low cost, and high safety. POMs exhibit unique structural characteristics and can be compatible with various oxygen sources, demonstrating excellent catalytic performance in oxidation reactions under mild conditions. However, POMs are easily soluble in polar solvents and function as homogeneous catalysts for the catalytic oxidation of fuels, leading to challenges such as complex product separation and catalyst recovery. To address these issues, introducing organic cations to form POM-IL in the POM catalytic system to adjust the solubility of POMs is a viable strategy, making POM-IL a promising oxidative desulfurization catalyst. Nevertheless, the direct oxidation of organic fuels by O_2_ faces a significant energy barrier, typically requiring high-temperature and high-pressure conditions or the presence of sacrificial agents. To tackle this challenge, a POM-IL system consisting of Anderson-type POMs and deep eutectic solvents (synthesized from polyethylene glycol and benzenesulfonic acid) was developed based on the biomimetic catalytic oxidation mechanism [47]. The POM-IL system [PyPS]CoMo (PyPS = 3-(pyridine-1-ium-1-yl) propane-1-sulfonate, CoMo = Co(OH)_6_Mo_6_O_18_^3−^) can be utilized for aerobic oxidative desulfurization under mild conditions (Figure 2a) [48]. It demonstrates outstanding catalytic performance, stability, and synthetic applicability. The remarkable oxidation efficiency stems from the unique catalytic mechanism. Cyclic voltammogram curves indicate that the peak current is 28 μA in the DESs and O_2_ systems, suggesting minimal O_2_ consumption near DESs. Conversely, in the presence of POMs, DESs, and O_2_, the peak current significantly increases from 17 to 470 μA, which indicates a large amount of O_2_ depletion in the vicinity of the electrode, confirming the high catalytic effect of DES (Figure 2b). In this process, Anderson-type POMs serve as the electron transfer mediator, facilitating electron transfer from POMs to DESs through a low-energy pathway. This reduces the activation energy of the desulfurization reaction, leading to exceptional diesel desulfurization performance (Figure 2c).

In the process of catalytic oxidative desulfurization, in addition to O_2_, H_2_O_2_ can also be used as an oxidizing agent. However, H_2_O_2_ is easy to decompose when heated, which not only reduces the utilization rate but also increases the reaction cost. Therefore, it is valuable to conduct a desulfurization reaction at room temperature. With this purpose, a series of POM-IL, [C*_n_*MIM]_5_VW_12_O_40_Br (*n* = 4, 8, 16, MIM = 1-alkyl-3-methylimidazolium) with different alkyl chain lengths were synthesized [49]. [C_6_MIM]_5_VW_12_O_40_Br has the best performance with a dosage of 10 mg and 100% conversion and selectivity in 50 min, as well as a maximum of 23 cycles at room temperature. This excellent catalytic performance is achieved since the long alkyl chain can enrich the reactant sulfides with similar polarity and repel sulfone products with large polarity. This “polar strategy” can not only accelerate the oxidative desulfurization reaction but also promote the enrichment and utilization of high-value-added sulfones.

In addition to the high conversion and selectivity, the good separation efficiency and recovery of catalysts from homogeneous reaction systems are also important. One efficient strategy to solve this problem is to load POM-IL on carriers. Through covalent binding, the prepared [C*_n_*MIM]_5_PMo_10_V_2_ (*n* = 4, 8, 12, 16) with different chain lengths are loaded on amine-modified magnetic graphene oxide (GO), and the hydrophilicity and hydrophobicity of the catalyst are adjustable by POM-IL and alkyl chain lengths [50]. The results indicate that the sample with an alkyl length chain of C_12_ has the highest dibenzothiophene (DBT) removal rate, which enables complete desulfurization of the model fuel. In addition, the catalytic system only needs to use permanent magnets to achieve emulsion demulsification and catalyst recovery.

The oxidative desulfurization process using POM-ILs as catalysts can be influenced by various factors. For instance, to study the impact of water on the catalytic reaction, a POM-IL system consisting of H_3_PM_12_O_40_ (PMo) and poly-[3-dodecyl-1-vinylimidazolium] bromine is constructed [51]. Experiments indicated that the catalytic conversion rates of DBT rose from 33.6% to 100% with the increase in water content from 0 to 70% by weight. This significant enhancement is attributed to the alteration of entropy enthalpy compensation in the presence of water, facilitating the transportation of H_2_O_2_ and thereby enhancing the catalytic activity. The state of existing cations is also a crucial factor for the performance of POM-ILs in catalytic reactions. When monomers, dimers, and polymer-[Vim] (Vim = 1-vinyl-3-amylimidazolium) cations interact with polyanions, the POM-ILs obtained on GO exhibit different desulfurization effects. Among them, P[Vim]POM shows the most effective performance in completely removing DBT within 1 h [52]. The choice of loading materials also plays a significant role. When the same POM-IL is loaded onto activated carbon, carbon aerogel, carbon nanotubes, and graphene oxide, POM-IL loaded on activated carbon shows the highest activity. This can be attributed to its wide specific surface area and the synergy between anions and cations [53]. Additionally, the catalytic oxidative desulfurization efficiency of POM-ILs can be influenced by the carbon chain length of POM-ILs [54,55] and the performance of hydrophilic, lipophilic, and amphiphilic [56].

Though several POM-ILs have been utilized in oxidative desulfurization, there is still a need to enhance the catalytic performance to achieve a shorter reaction time, higher transformation rate, and milder reaction conditions. These objectives can be achieved by exploring various types of polyanions that have not been previously utilized in this process, as they can serve different functions in reactions to enhance catalytic efficiency.

### 2.2. Alcohol Oxidation

**Figure 3:**
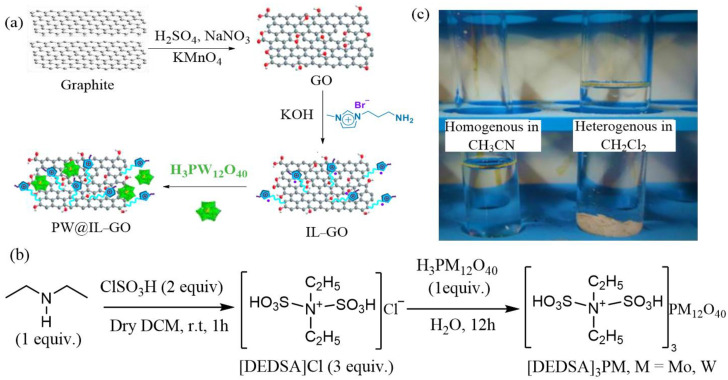
Synthesis route of (**a**) PW@IL-GO. Reproduced with permission from [59]. Copyright (2020) Elsevier. (**b**) [DEDSA]_3_PM. (**c**) Catalysis in CH_3_CN and CH_2_Cl_2_. Reproduced with permission from [60]. Copyright (2020) Elsevier.

In addition to oxidative desulfurization, POM-ILs can also be used in alcohol reactions to prepare aldehydes or ketones, which are important synthetic intermediates in the pharmaceutical and chemical industries [57]. Adhering to the principles of green chemistry, hydrogen peroxide is usually chosen as an oxidant to oxidize alcohols to obtain carbonyl compounds [58], for example, through a ring-opening reaction of the amino group of the amine-functionalized imidazole IL (IL-NH_2_) and the GO epoxy group to synthesize IL-GO by the combination of the curing method and immobilization method. A variety of heterogeneous catalysts PW@IL-GO(m) (m represent the dosages of PW and are 0.5, 1.0, and 1.5 mmol, respectively) are prepared through the anion exchange and ammonia protonation reaction (Figure 3a) [59]. This preparation method improves the dispersion, accessibility, and stability of the active site. The amphiphilia of IL-GO flakes and the porosity of PW@IL-GO(m) are beneficial for reducing mass transfer limitations in aqueous/organic matrix two-phase systems. The experimental results show that PW@IL-GO(1.5) has a maximum conversion of 94% for benzyl alcohol and a selectivity of 91% for benzaldehyde. In addition to the high conversion rate and selectivity, recyclability is also an important issue. The simple recovery of catalysts can be achieved by loading POM-ILs onto a support with a high surface area. However, due to the weak force between the support catalysts, there is a problem of slow aggregation and leaching reaction rates on the support. To overcome this shortcoming, a different strategy is applied. As an example, two POM-IL catalysts, [DEDSA]_3_PM (M = Mo, and W, [DEDSA] = diethyldisulphoammonium), are prepared by a two-step method using diethyldisulphoammonium chloride [DEDSA]Cl, PMo, and PW (Figure 3b) [60]. Under optimal reaction conditions, the catalytic oxidation of phenylethyl alcohol is carried out, and the yield of acetophenone reaches 98%. Since the cation in the catalyst contains a sulfonic acid group, it forms hydrogen bonds with anions and solvent molecules. This reduces the difficulty of separating the solvent from the catalyst and enables the transition between homogeneous and heterogeneous catalysis. The different effects of catalysts on different solvents result in different solubilities. Catalytic alcohols are oxidized under homogeneous conditions, and the catalyst is precipitated after the addition of the CH_2_Cl_2_ solvent after the reaction to achieve solvent-responsive self-separation, which has the advantages of high homogeneous catalytic activity, heterogeneous catalytic recovery, and easy recovery (Figure 3c).

POM-ILs have been applied to the catalytic oxidation of alcohols under the condition that H_2_O_2_ is used as an oxidant, while POM-IL as a catalyst has the problem of recovery, which limits its wide application. In the future, POM can be modified by adding specific organic functional groups to make it have self-separation performance and obtain catalysts that respond to specific additives, temperatures, solvents, or other irritants so as to improve the recovery rate of POM-IL catalysts and improve the catalytic performance.

### 2.3. Olefin Epoxidation

**Figure 4:**
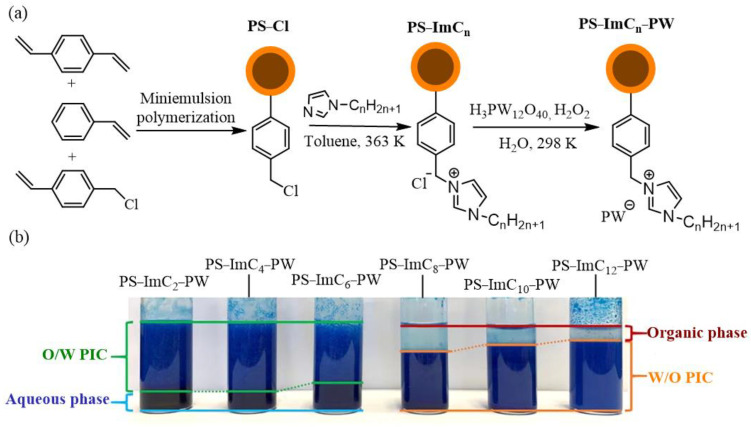
(**a**) Synthesis procedure and structure of the polymer PS-ImC_n_-PW. (**b**) Optical images of the allyl chloride/H_2_O_2_ Pickering interfacial catalytic systems stabilized by various catalysts (Pickering interfacial catalytic systems are stained with methylene blue, standing for 1 h after vigorous shaking). Reproduced with permission from [62]. Copyright (2023) American Chemical Society.

Olefin epoxidation is a crucial method in chemical synthesis. For instance, the reaction between chloropropylene and H_2_O_2_ to produce epichlorohydrin is widely used in industry due to its environmental friendliness. Epichlorohydrin has various applications in epoxy resins, synthetic glycerin, and chlorohydrin rubber, serving as a solvent, surfactant, and plasticizer. However, the reaction is hindered by mass transfer issues at the liquid–liquid interface, leading to low reaction rates. Increasing the H_2_O_2_ concentration or temperature for faster reactions compromises safety and control. To address this challenge, a Pickering interfacial catalytic system is developed. Pickering emulsions not only enhance reaction rates by overcoming mass transfer limitations but also enable the easy recovery of heterogeneous catalysts [61]. Following this approach, polymer nanospheres with cross-linked polystyrene as the core and POM-IL as the shell are synthesized by grafting POM-IL onto core-shell nanospheres (Figure 4a) [62]. Nanospheres can emulsify chloropropylene and H_2_O_2_, with the carbon chain length of the IL in the shell influencing the type of Pickering emulsion (Figure 4b). C_2_–C_6_ results in O/W-type emulsions, while C_8_–C_12_ lead to W/O-type emulsions. Experimental findings indicate that PS-ImC_2_-PW and PS-ImC_12_PW are the most effective catalysts in O/W and W/O type emulsions, respectively. Using 45 mg of PS-ImC_2_-PW with a water/oil volume ratio of 1.17/0.41, the conversion rate of chloropropylene reaches 85.4% after 4 h, with a turnover frequency value of 81.8 h^−1^. Similarly, with the same dosage of PS-ImC_2_-PW, the water/oil volume ratio is 1.17/0.41, the H_2_O_2_ is 9.6wt% as the oxidant, the conversion rate is 70.8%, and the turnover frequency value is 67.8 h^−1^. Furthermore, the reaction is conducted under solvent-free conditions, making it convenient for product separation. Upon completion of the reaction, the catalyst is recovered and reused for eight cycles, demonstrating its excellent stability.

The key to enhancing the conversion rate of olefin epoxidation lies in choosing an effective catalytic system, like POM and titanium–silicon zeolite catalysts for propylene epoxidation. It is essential to investigate a solvent-free catalytic system for olefin epoxidation reactions.

### 2.4. Oxidative Degradation of Herbicides

In order to meet the global demand for food and ensure the growth and yield of crops, more and more herbicides are used; however, they have also caused great harm to the environment. For example, metobromuron is a common herbicide, and it has a low absorption rate in the soil and high water solubility, which leads to problems such as pollution of aquatic reservoirs, deterioration of water quality, etc. Therefore, the degradation and removal of toxic pollutants become an important challenge. To degradation of herbicides, two POM-ILs with the same organic cation, [DBDSA]^+^ ([DBDSA]^+^ = dibutyldisulfoammonium), and different Keggin-type clusters PM are built and named as [DBDSA]_3_PM [63]. The prepared POM-IL is insoluble in water and is used as a heterogeneous catalyst for the degradation of herbicides in aqueous media. With H_2_O_2_ as an oxidant, the total organic carbon removal rate can reach 70% after 8 h of sunlight irradiation. Furthermore, 1-bromo-4-isocyanatobenzene is generated, which is the main value-added product for the synthesis of various unsymmetrical disubstituted urea derivatives.

## 3. The Applications of POM-ILs in the Lysis Reaction

As high-efficiency catalysts, ILs have shown excellent performance in both oxidation and cracking catalytic reactions. While nanospheres and metal salts are commonly used as catalysts for degradation reactions, they often suffer from issues such as low reaction rates and instability. On the other hand, POMs exhibit good molecular structures and stability, possessing acid–base and redox capabilities. When combined with ILs to form POM-ILs through ion exchange, they result in catalysts with high catalytic activity and easy recovery. This section explores the use of POM-ILs as catalysts in pyrolysis reactions.

### 3.1. Degradable Polyethylene Terephthalate

**Figure 5:**
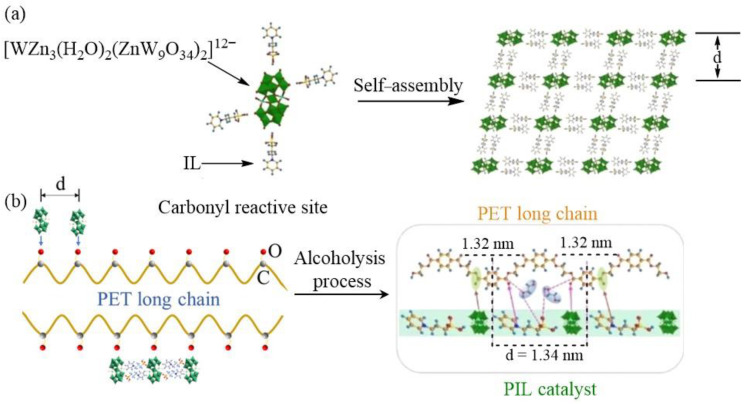
(**a**) Schematic illustration of the possible crystal structure of the prepared POM-IL. (**b**) Pore diameters of PET alcoholysis solid products with or without catalysts. Reproduced with permission from [66]. Copyright (2023) John Wiley and Sons.

Polyethylene terephthalate (PET) is a beneficial thermoplastic material extensively used in food packaging. However, while PET offers convenience, it also contributes to environmental pollution, making the recycling and degradation of PET a pressing issue. Chemical degradation methods for PET include hydrolysis and alcoholization [64]. The hydrolysis process requires high-temperature and high-pressure conditions, leading to high costs. As a result, the alcoholization method has garnered significant attention. To address this, five POM-ILs, BMIM_x_[M(H_2_O)TiMo_11_O_39_] (BMIM = 1-butyl-3-methylimidazolium, M = Cu^2+^, Fe^2+^, Pb^2+^, Zn^2+^, x = 6; M = Ti^4+^, x = 4), were synthesized using Ti-centered POM with various metal vacancies and ion-exchanged with 1-butyl-3-methylimidazolium chloride IL [65]. These POM-ILs were utilized to catalyze the degradation of bottle-grade PET, with BMIM_4_[Ti(H_2_O)TiMo_11_O_39_] exhibiting the most effective catalytic performance, achieving nearly 100% degradation in 5 h and enabling at least six cycles of recyclability. Despite the high degradation rate, the reaction time remains somewhat lengthy. One efficient approach to enhance reaction efficiency is to achieve nanoscale precision cutting of the PET chain quickly by precisely designing the catalyst. To this end, pyridinium (Py), PyPs, 1-(3-sulfonic group) triethylamine (TEAPs) and 1-methyl-3-(3-sulfopropyl)imidazolium (MIMPs) ILs were chosen to combine with [WZn_3_(H_2_O)_2_(ZnW_9_O_34_)_2_]^12−^ to produce POM-ILs (Figure 5) [66]. All of these catalysts, X*_n_*[WZn_3_(H_2_O)_2_(ZnW_9_O_34_)_2_] (X = PyPs, Py, MIMPs, TEAPs, *n* = 3, 6, 9, 12), exhibit high thermal stability, with controlled interlayer distances ranging from 1.00 to 1.63 nm. When the molar ratio of anion to cation is 1:6, the interlayer distance of the catalyst is 1.34 nm, aligning the spacing of the active sites with the carbonyl spacing at both ends of the benzene ring in PET (1.32 nm). With this strategy, PET can be completely degraded within 0.5 h, demonstrating that the precise cutting of the PET chain plays a crucial role in the catalytic reaction.

### 3.2. Lysis of Lignocellulose

**Figure 6:**
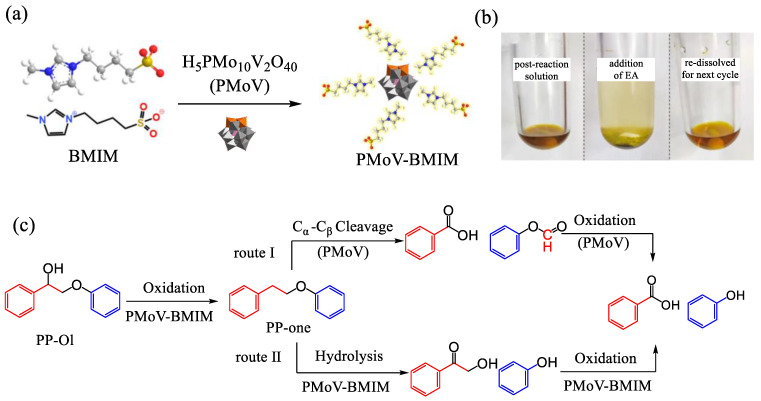
(**a**) Synthetic route of PMoV-BMIM. (**b**) Digital photographs of the reaction solutions catalyzed by the PMoV-BMIM before (left), after the addition of ethyl acetate (middle), and re-dissolving for the next cycle (right). (**c**) Proposed pathways for the cleavage of PP-ol by PMoV-BMIM. Reproduced with permission from [70]. Copyright (2023) The Royal Society of Chemistry.

In addition to polymer materials, POM-ILs are also applicable in the degradation of natural materials. Lignocellulose, the most abundant renewable biomass on Earth, consists of 35–50% cellulose, 15–30% lignin, and 25–30% hemicellulose [67]. These components can be utilized for the production of biochemical products and biodiesel, thus helping to alleviate energy stress and environmental pollution [68,69]. POMs have been integrated into ILs due to their unique acidic properties, which can offer acidic sites for cellulose cleavage to achieve high yields of value-added products. To convert lignocellulose into more valuable chemicals, PMoV-BMIM (PMoV = H_5_PMo_10_V_2_O_40_) catalysts are created by combining PMoV and zwitterion BMIM through electrostatic interactions (Figure 6a) [70]. The resulting PMoV-BMIM catalyst is then employed to catalyze various β-O-4 lignins, leading to the conversion of 2-phenoxy-1-phenylethanol into benzoic acid and phenol with yields of 96% and 80%, respectively. Moreover, the strong bond between the inorganic and organic components enables easy separation of the catalyst by adding ethyl acetate to the reaction system. The precipitated catalyst can be reused at least five times without a significant decrease in catalytic activity (Figure 6b). Mechanistic studies suggest two proposed reaction pathways in the catalytic process involving the same initial oxidation reaction and subsequent different transformations (Figure 6c).

It is insufficient to convert only a portion of the lignocellulose composition into a value-added product. To enhance the utilization of lignocellulose, it is essential to develop a catalyst capable of converting all the primary components of lignocellulose into the desired product. To achieve this, a Cu-centered POM-IL, [BSMIM]CuPW_12_O_40_ (BSMIM = butyl sulfonate-3-methylimidazolium), was chosen to create a catalyst [71]. When using this catalyst with ethanol as a solvent, cellulose, lignin, and hemicellulose can all be transformed into diethyl maleate, a product in high commercial demand. The yield of diethyl maleate reaches 320 mg/g with a selectivity of up to 70%. This remarkable catalytic performance is attributed to the presence of a small amount of Cu^2+^ in [BSMIM]CuPW_12_O_40_, which offers vacant orbitals for oxygen molecules. The polyanion plays a synergistic role, while the catalytic oxidation site and acid site are crucial in the catalytic process. Moreover, the catalyst can be easily recovered and reused up to six times without a significant loss of catalytic activity.

Therefore, the combined effect of POM and IL can be utilized to degrade lignocellulose with various benefits, including enhancing the solubility of lignocellulose, reducing the formation of by-products, and improving the efficiency of catalyst recovery. However, there is still potential for enhancing the conversion rate of desired products and achieving accurate transformation. The current predominant approach involves incorporating more active sites in POM-IL to enable precise transformation at the nanoscale, which will be the focal point of future advancements in this field.

## 4. The Applications of POM-ILs in Other Catalytic Reactions

POM-ILs can be formed using different organic cations and various POMs, enabling high proton mobility, multi-electron redox activity, and adjustable solubility. Additionally, the molecular structure of POM anions and IL cations can be tailored to bridge the gap between homogeneous and heterogeneous catalysis. As a result, POM-ILs can serve not only as catalysts for oxidation and cleavage reactions but also find extensive applications in catalyzing various other reactions like formylation and esterification and in the production of valuable compounds such as aspirin, paracetamol, and more.

### 4.1. Formylation

**Figure 7:**
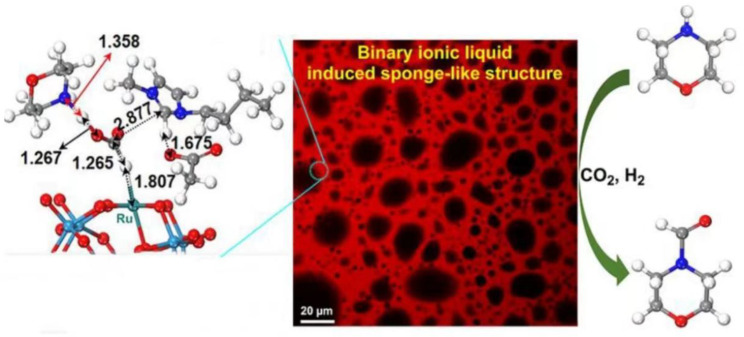
Laser confocal scanning microscopy image of a three-component (amine, Ru-POM-IL, and BMIMOAc) system and its catalysis of *N*-formylation of amines with CO_2_ and H_2_. The unit for the numbers in the left figure is Å. Reproduced with permission from [75]. Copyright (2023) American Chemical Society.

CO_2_ is widely available, safe, inexpensive, and abundant in nature, and the carbonylation reaction of CO_2_ with organic amines is a cost-effective method for producing formamide, a high-value-added product [72]. However, the conversion of CO_2_ involves three steps: adsorption, activation, and conversion. ILs can serve not only as the reaction medium but also as agents for adsorbing and activating CO_2_, as well as facilitating the separation of catalysts for recycling and reuse. Previous studies have shown that Ru^II^ complexes can enhance the catalytic activity of the N-formylation reaction of amines [73], but they require expensive organic ligands to stabilize the active center of Ru. These complexes are used as homogeneous catalysts, making it challenging to recover and reuse them after the reaction. POMs have well-defined structural features and anchor anchors that can be used as carriers for Ru. This effectively reduces the aggregation between individual metal atoms and retains the catalytic active center to a large extent [74]. [TOMA_6_SiW_11_O_39_Ru(DMSO)], abbreviated as Ru-POM-IL, and TOMA as methyltrioctylammonium, is successfully prepared using the hydrothermal method and two-phase ion exchange method [75]. In the presence of BMIMOAc, it efficiently catalyzes the formylation reaction of various primary and secondary amines with CO_2_ and H_2_, resulting in a high formamide yield (Figure 7). The structure of Ru-POM-IL-BMIMOAc is sponge-like, with BMIMOAc in the polar region and Ru-POM-IL in the non-polar region, enhancing the contact area between the substrate and the catalytic active center. This configuration aids in the adsorption and activation of CO_2_. Through in situ IR experiments and density functional theory calculations, it is demonstrated that the catalyst functions as a three-component synergistic system in the formylation reaction of amines with CO_2_ and H_2_. The synergy among the components reduces the high reaction energy barrier during CO_2_ conversion, enabling efficient catalysis and improving reaction efficiency and yield. The catalyst exhibits excellent recycling capabilities and can remain stable in the air for extended periods.

Single-atom catalysts can not only facilitate the comprehension of catalysis principles at the atomic level but also enhance the atomic efficiency of the central metal atom and decrease synthesis costs. The distinctive structural features of POMs provide anchor sites, making the loading of single-atom catalysts on POM-IL a viable solution for efficiently catalyzing formylation reactions.

### 4.2. Esterification Reaction

**Figure 8:**
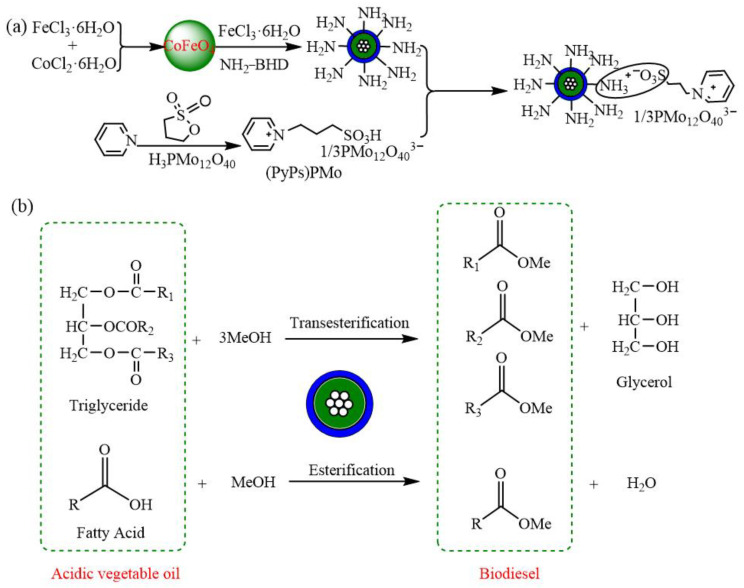
(**a**) Schematic illustration for the preparation of CoFe_2_O_4_/MIL-88B(Fe)-NH_2_/(PyPs)PMo, and (**b**) its utilization as a catalyst for the transformation of triglycerides and free fatty acids. Reproduced with permission from [79]. Copyright (2020) Elsevier.

Biodiesel can serve as a substitute for traditional fossil fuels and is often produced through the esterification of vegetable oils, animal fats, or waste cooking oil. The use of low-quality oils and fats, such as waste cooking oil, has garnered attention for its potential to reduce production costs and reuse waste. However, the presence of a large amount of free fatty acids and water in waste cooking oil can lead to issues with alkaline catalysts, causing a loss of an active center, reduced catalytic effect, and emulsification, making product separation difficult [76]. These challenges can be overcome by utilizing acid catalysts, which can also facilitate transesterification to produce biodiesel simultaneously [77]. POMs are an excellent choice for the preparation of acidic catalysts due to their outstanding acidity and catalytic capacity [78]. For example, POM-IL (PyPs)PMo is synthesized from PMo using an anion-exchange method with pyridine and 1,3-propanesultone as raw materials. The final product, CoFe_2_O_4_/MIL-88B(Fe)-NH_2_/(PyPs)PMo, is prepared by electrostatic loading on the amino-functionalized metal–organic framework (CoFe_2_O_4_/MIL-88B(Fe)NH_2_) (Figure 8a) [79]. The catalyst is utilized for the one-pot synthesis of biodiesel from low-quality oil. It possesses Brønsted–Lewis acidic sites. The combination of POM-IL and the porous material MOF not only enhances the contact area between the catalyst and the substrate but also prevents the aggregation of magnetic particles, significantly improving the catalytic efficiency. Complete conversion of free fatty acids can be achieved after 8 h of reaction, and the catalytic performance of the catalyst in converting free fatty acids to diesel surpassed that of transesterification reactions. The catalytic mechanism of the solid acid is proposed. Initially, the catalyst interacts with the carbonyl group in triglycerides or free fatty acids to form carbocations, enhancing its electrophilic ability. Subsequently, ethanol attacks the carbocation nucleophilically to produce unstable intermediates. Finally, electrons are transferred, and chemical bonds are broken to yield fatty acid methyl esters. Due to the superparamagnetism of the catalyst, it can be entirely recovered using permanent magnets after the reaction (Figure 8b).

It is crucial to discover green and cost-effective solid catalysts for bi-diesel production. However, there are obstacles hindering their application, such as high costs and complex synthesis processes. Overcoming these shortcomings is a key focus in the field of solid catalysts.

### 4.3. Catalytic Synthesis of Aspirin and Paracetamol

**Figure 9:**
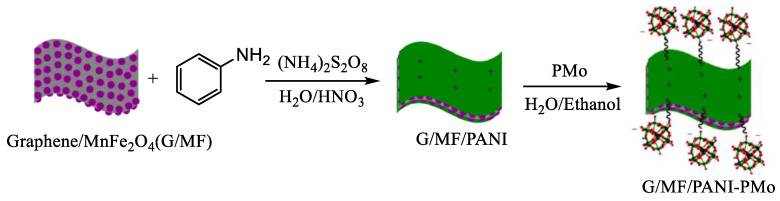
Synthetic route of the G/MF/PANI-PMo nanocatalyst. Reproduced with permission from [83]. Copyright (2021) John Wiley and Sons.

Aspirin (acetylsalicylic acid) and paracetamol (*N*-(4-hydroxyphenyl)acetamide) are key components of analgesic medications in the medical field and crucial precursors in organic synthesis. Therefore, the efficient synthesis of aspirin and paracetamol holds significant importance [80,81]. Sulfuric acid is commonly utilized as a catalyst in this process, but it is highly corrosive, damages equipment, incurs high synthesis costs, and results in low yields. In contrast, POM has emerged as a solid catalyst with Brønsted acid properties, garnering considerable attention. Magnetic nanocatalysts, known as green catalysts, have also become prominent in catalytic reactions due to their extensive surface area and numerous catalytic sites. These catalysts can effectively interact with precursors and be easily separated from reaction systems using external magnetic fields, combining the benefits of both homogeneous and heterogeneous catalysis [82]. To this end, manganese ferrite nanoparticles are deposited onto graphene surfaces, followed by an even coating of polyaniline (PANI) and functionalization with PMo to produce G/MF/PANI-PMo (Figure 9) [83]. In this catalytic system, graphene offers a large surface area, PMo contributes to Brønsted acidic sites, manganese ferrite provides magnetism, and PANI immobilizes PMo. Under optimal reaction conditions, the solvent-free synthesis of aspirin and paracetamol using the G/MF/PANI-PMo catalyst exhibited high catalytic activity and selectivity, yielding 97% aspirin and 98% paracetamol.

Compared to other catalysts, POM-IL can efficiently catalyze the synthesis of aspirin and paracetamol quickly under conditions like room temperature and pressure, solvent-free environments, and other simple conditions. This method avoids the need for complex compounds and expensive instruments, offering advantages in terms of time, cost, and safety. It represents a promising approach for the large-scale production of aspirin and paracetamol.

## 5. The Applications of POM-ILs in Adsorption

POM exhibits a robust load-bearing capacity and the ability to release electrons without altering its structure, demonstrating exceptional redox capability. Additionally, POM showcases outstanding adsorption performance [84,85] and serves as an environmentally friendly inorganic material widely utilized in wastewater treatment, purification, handling toxic gases, and addressing other environmental issues. IL, characterized by low toxicity, high stability, and low vapor pressure, is recognized as a green solvent with a modifiable structure, finding applications in various hybrid materials. As a result, POM-IL can serve not only as a catalyst for diverse chemical reactions but also possess a certain adsorption capacity for dyes and biological proteins in sewage.

**Figure 10:**
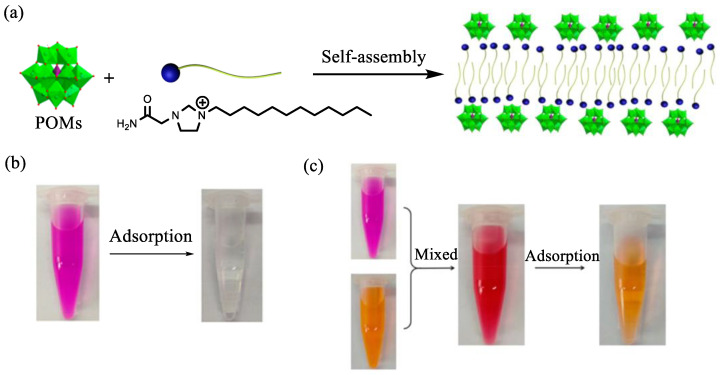
(**a**) Synthetic route and structure of SAILLEP. (**b**) Optical images of rhodamine B solution before and after the adsorption by SAILLEP. (**c**) Optical images of the mixture of rhodamine B and eosin Y before and after adsorption by SAILEP. Reproduced with permission from [87]. Copyright (2019) American Chemical Society.

POM-IL (BMIM)_3_PW is prepared by reacting Keggin-type PW with BMIMBr at room temperature. Subsequently, the POM-IL encapsulated ZIF-8-(BMIM)_3_PW is synthesized by the reaction of POM-IL with ZIF-8. The modification of ZIF-8 by POM-IL significantly enhances the adsorption capacity of ZIF-8 for the cationic dye methylene blue [86]. With an adsorbent concentration of 0.04 g∙L^−1^ and a pH of 11, the maximum removal rate of the cationic dye MB reaches 95.75% after 0.75 h of reaction. Besides its exceptional adsorption capacity for MB, POM-IL also exhibits similar properties towards other dyes. For instance, surfactant SAILEPs are created through the self-assembly of PW and surface-active IL *N*-dodecyl-*N*′-acetamideimidazolium bromide at room temperature (Figure 10a) [87]. The adsorbent demonstrated low adsorption capacity for anionic dyes but showed efficient adsorption capacity for cationic dyes, particularly rhodamine B, achieving adsorption equilibrium within just 60 s when mixed with RhB. Due to the strong affinity and electrostatic effect of POM on RhB, surface-active ionic liquid-encapsulated polyoxometalates (SAILEPs) can selectively separate RhB from mixed solutions of eosin Y and rhodamine B (Figure 10b,c). The adsorption kinetics, adsorption isotherms, and adsorption mechanisms indicate that the adsorption process aligns with the Langmuir isothermal model and the quasi-second-order kinetic model, with SAILEPs facilitating the monolayer adsorption of cationic dyes.

POM-ILs can not only adsorb dyes to purify industrial wastewater but also separate proteins through protein adsorption, providing a new perspective for the construction of bio-analytical composites. *N*,*N*-dimethyl-dodecyl-(4-vinylbenzyl) ammonium chloride (DDVAC) is prepared by free radical polymerization to create the polyionic liquid PDDVAC, and the porous Co_4_PW-PDDVAC (Co_4_PW = Na_10_[Co_4_(H_2_O)_2_(PW_9_O_34_)_2_]) is prepared by a cation exchange method using PDDVAC and Co_4_PW as raw materials [88]. Interestingly, when adjusting the molar ratios of POMs and ILs, composites with different structures are obtained, but they face some issues, such as uneven pore size distribution. There is a strong covalent coordination and hydrogen bonding between Co^2+^ and proteinase K in Co_4_PW, which gives the Co_4_PW-PDDVAC composites an excellent adsorption capacity for proteinase K. The thermodynamic results show that the adsorption process follows the Langmuir isotherm with a capacity of up to 1428 mg∙g^−1^. When an imidazole solution is used as a dissolving agent, the Co_4_PW-PDDVAC composite can selectively separate high-purity PrK from complex matrices.

POM-ILs offer the unique advantage of rapidly adsorbing target substances, making them suitable for dye adsorption in sewage treatment and protein separation in biological analysis. They have a wide range of potential applications in fast, cost-effective, and high-throughput adsorption systems. However, the presence of POM-IL, such as uneven pore size distribution, can significantly impact adsorption performance. Therefore, it is crucial to identify a suitable POM-IL system and design composite materials with a clear structure and uniform particle size.

## 6. The Applications of POM-ILs in Lithium-Ion Batteries

POM-IL can also be applied in the battery field. POM is a promising material for batteries because of its unique physical and chemical properties, such as strong electron acceptance, photo-sensitivity, and stability. These properties can be precisely tailored by modifying metal ions, heteroatoms, and counterions. On the other hand, IL is commonly used for energy conversion and storage due to its non-flammability, low volatility, and diverse structure. Therefore, combining POMs and ILs is an effective approach for developing battery materials. This section explores the use of POM-IL in the battery field.

**Figure 11:**
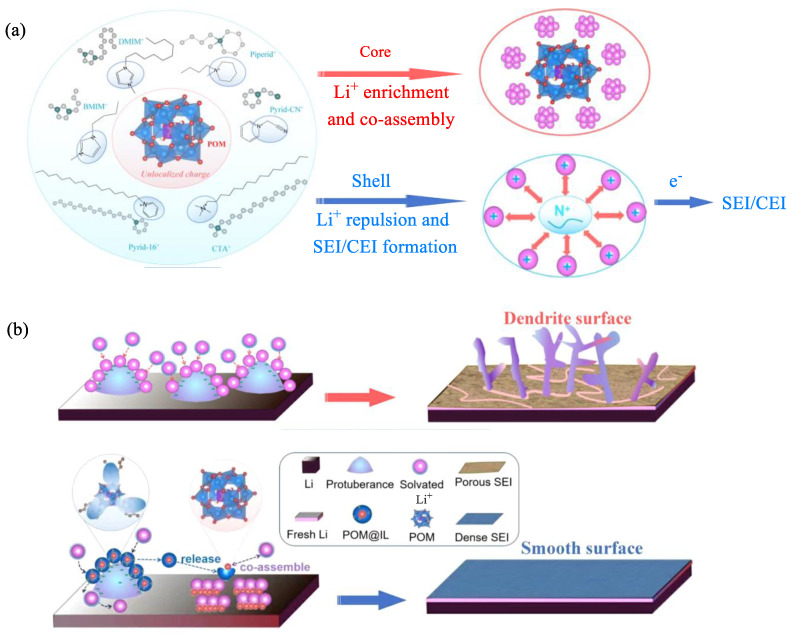
(**a**) The diagram shows the preparation of POM@BMIM and its functional components. (**b**) Comparison of dendrite growth conditions on solid electrolyte interface with and without POM@BMIM. Reproduced with permission from [91]. Copyright (2021) John Wiley and Sons.

Lithium metal is a highly promising material for developing next-generation high-energy-density battery anodes due to its ultra-high theoretical capacity and extremely low electrochemical potential. However, the progress of lithium-ion batteries has been hindered by issues like irreversible lithium loss and dendrite growth [89]. The properties and composition of the solid electrolyte interface can be controlled by adding suitable electrolyte additives to effectively inhibit dendrite growth [90]. Therefore, a group of “core-shell” additives with Keggin-type POM as the “core” and ILs with various nitrogen-containing cations as the “shell” were prepared [91]. Cations BMIM^+^, 1-decyl-3-methylimidazolium, 1-butyl-1-methylpiperidinium, and cetylpyridinium, 1-(cyanomethyl)pyridinium, hexadecyl trimethylammonium, and a polar PEG polymer are combined with PW and PMo to create a new class of additives for stabilizing the Li/electrolyte and electrolyte/NCA (NCA = LiNi_0.8_Co_0.15_Al_0.05_O_2_) in a lithium-ion battery (Figure 11a). POM@BMIM is easily adsorbed at the protruding tip in the ether-based electrolyte solution; BMIM^+^ as a “shell” exerted an electrostatic shielding effect, repelling Li^+^ to redistribute it away from the tip and ensuring even distribution. Subsequently, the POM anion, acting as the “nucleus,” is gradually released and becomes the center of Li^+^ enrichment, with POM and Li^+^ co-assembling after capturing electrons. The repulsion-enrichment synergy of Li^+^ between IL and POM adjusts the Li^+^ deposition behavior, leading to uniform deposition and improved solid electrolyte interface (Figure 11b). This additive enables Li||Li symmetrical batteries to have a lifespan exceeding 500 h and 300 h at high current densities of 3 and 5 mA cm^−2^, respectively. Furthermore, POM@CTA sustained-release additives are utilized in combination with Li||NCA battery compatibility, maintaining a specific capacity of over 100 mAh g^−1^ at 1 C for a minimum of 200 cycles. Even when the positive electrode loading reaches 20 mg cm^−2^ at 2.6 mA, Li||NCA batteries can still be cycled over 100 times with a specific capacity exceeding 100 mAh g^−1^.

In lithium-ion batteries, POM-IL can enhance the deposition of Li^+^ and facilitate more Li^+^ participation in the intercalation/deintercalation process of lithium-ion batteries. The charging and discharging of lithium-ion batteries involve lithium embedding and deintercalation between the electrodes. Reduced GO (RGO) and POM composites offer a high specific surface area, providing additional Li^+^ storage sites and facilitating electrolyte penetration and Li^+^ diffusion for enhanced Li^+^ embedding and deintercalation. The nanocomposite PMo@RGO-AIL, formed by covalently bonding RGO and PMo as the cathode of the lithium-ion battery, exhibits an initial discharge capacity of 730.2 mAh g^−1^ at a current density of 50 mA g^−1^. Even after 100 cycles, the specific capacity remains high at 472.6 mAh g^−1^, demonstrating the excellent cycling stability of PMo@RGO-AIL [92].

Due to the unique structure of POM and IL and the distinct properties they possess, POM-ILs demonstrate a wide range of potential applications in the battery field. These systems can be utilized to create electrode materials and electrolytes that can cater to various requirements in the future.

## 7. The Applications of POM-ILs in Antibacterial

The combination of IL and POM is widely used in biological fields, such as antibacterial. IL’s antimicrobial activity is linked to its lipophilic/hydrophobic properties, enabling interactions with fungal and bacterial cell walls. POM is biologically active due to its ability to interact with protein amino acids, leading to reactions with cellular bacteria that impact their viability. This section will concentrate on the use of POM-ILs in antimicrobial applications.

**Figure 12:**
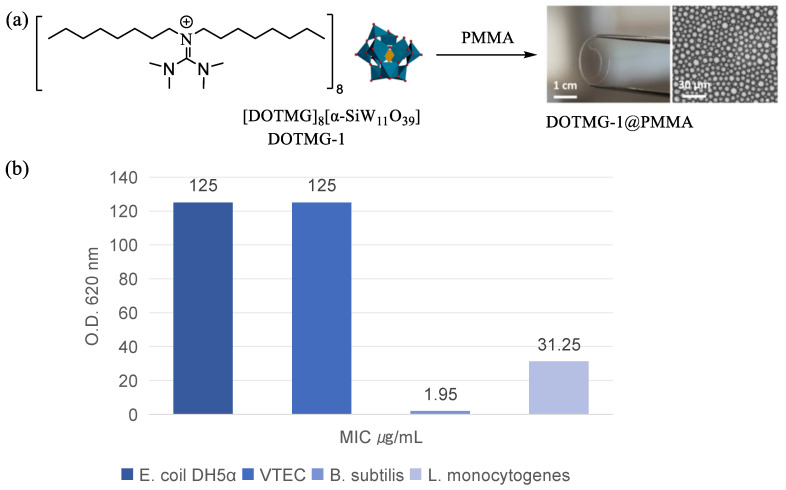
(**a**) Synthetic route and structure of DOTMG-1@PMMA. (**b**) Growth curves of E. *coli* DH5α, VTEC, B. *subtilis*, and L. *monocytogenes* in the presence of various concentrations of DOTMG-1. Reproduced with permission from [95]. Copyright (2022) American Chemical Society.

With the widespread use of antimicrobials, antimicrobial resistance in bacteria and fungi is increasing, leading to the need for new antimicrobial coating materials to prevent the spread of bacteria and fungi through human contact on surfaces like tabletops, handles, and food packaging bags [93,94]. Derivatives of guanidine are key components of well-known antimicrobial agents. Therefore, POM-IL DOTMG-1 is developed by combining IL *N*,*N*,*N*′,*N*′-tetramethyl-*N*″,*N*″-dioctylguanidinum (DOTMG) with monovacant Keggin-type POM SiW_11_, loaded onto poly(methylmethacrylate) (PMMA) to create an antibacterial film (Figure 12a) [95]. Tests on the antimicrobial properties of DOTMG-1 show that Gram-positive B. subtilis is the most sensitive microorganism with a minimum inhibitory concentration (MIC) of 1.95 μg mL^−1^ and a minimum bactericidal concentration of 3.91 μg mL^−1^ (Figure 12b), indicating the high bacteriostatic and bactericidal effectiveness of the prepared POM-IL. Mold A. niger demonstrates the highest resistance to DOTMG-1 with an MIC of 250 μg mL^−1^. The experimental results indicate that when the weight ratio of DOTMG-1 to PMMA is 20:80, the film can effectively inhibit bacterial growth and biofilm formation, making it suitable for surface coating or food packaging materials in healthcare settings to prevent the proliferation and survival of microorganisms.

The use of POM-IL in antimicrobial applications can safeguard human health and preserve cultural artifacts. For instance, certain fungi can generate melanin, which may harm the aesthetics and composition of cultural relics. To enhance the protection of cultural artifacts, three SiW_11_-based POM-ILs (SiW_11_ = [α-SiW_11_O_39_]^8−^) are synthesized with varying carbon chain lengths: [P_44412_][SiW_11_], [P_44414_][SiW_11_], and [P_66614_][SiW_11_] (P_44412_, P_44414_, and P_66614_ represent tribu-tyldodecyl, tributyltetradecyl, and trihexyltetradecyl, respectively) [96]. Four fungal species are chosen, and the antifungal efficacy of POM-ILs is assessed. All three POM-ILs exhibit strong antifungal properties, with [P_44412_][SiW_11_] displaying the most effective performance due to its shorter alkyl chain, suggesting that adjusting the alkyl chain length can enhance antifungal effectiveness.

Because of the features of low-cost and easy preparation of POM-ILs, they are expected to be used in the preventative bactericidal coating for calcareous stones. In a typical example, a POM-IL comprising SiW_11_, tetraheptylammonium, and trihexyltetradecylammonium is prepared, and its durability and performance of biocidal are evaluated by coating it on four types of limestones [97]. The results indicate that the coated POM-IL can prevent the colonization of algal biofilms with an unchanged color. It should be noted that the concentration of POM-IL must be carefully controlled to strike a balance between the porosity of the stone, the color change generated, and the desired biocidal effect over a long period.

Despite the application of numerous POM-IL systems in antibacterial contexts, their performance remains suboptimal primarily due to the prevalence of drug resistance, indicating the need for further exploration of more suitable POM-IL systems. Introducing IL cations with higher antimicrobial efficiency to bolster the broad-spectrum antibacterial activity of POM-IL and its associated materials is a key future development direction in this field. More simulated and real-world outdoor tests are carried out on the new POM-IL.

## 8. The Applications of POM-ILs in Other Areas

Due to the excellent characteristics, POM-ILs can be used in various applications such as anticorrosion protection and solar panels in addition to the above-mentioned fields.

### 8.1. Anticorrosion

**Figure 13:**
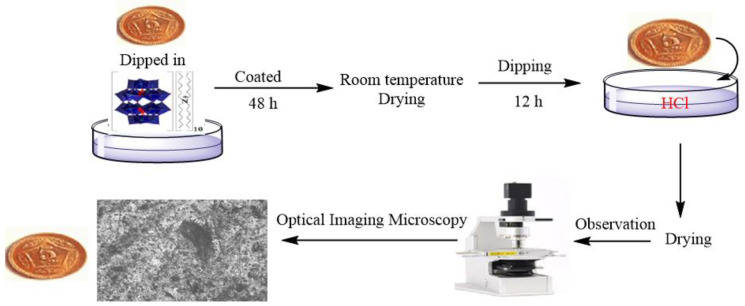
The procedure for coating coins with W_17_@POM-IL and V_3_W_15_@POM-IL and treating them with HCl. Reproduced with permission from [99]. Copyright (2023) Elsevier.

Corrosion is a significant natural phenomenon, causing severe damage to structures like buildings, statues, and cultural artifacts. Metal corrosion is particularly concerning, with approximately 3% of the gross domestic product affected each year [98]. To assess the effectiveness of POM-ILs in preventing corrosion, two POMs, K_10_[α_2_-P_2_W_17_O_61_]∙20H_2_O (P_2_W_17_) and K_8_HP_2_W_15_V_3_O_62_·9H_2_O (P_2_V_3_W_15_), are combined with (C_7_H_13_)_4_N^+^, generating two POM-ILs, P_2_W_17_-C_7_N and P_2_V_3_W_15_-C_7_N [99]. Acid corrosion tests reveal that coins immersed in 50% HCl for 48 h experience a mass loss rate exceeding 10%. In contrast, coins coated with P_2_W_17_-C_7_N and P_2_V_3_W_15_-C_7_N exhibit significantly lower weight loss rates of only 1.7% and 3.7%, respectively, under the same conditions, demonstrating the effective corrosion resistance of POM-ILs (Figure 13). Electrochemical impedance tests indicate that the charge transfer ratios for P_2_W_17_-C_7_N, P_2_V_3_W_15_-C_7_N, coated, and uncoated coins are 7.03, 5.23, and 3.78 Ω, highlighting the superior anticorrosion properties of POM-ILs. Additionally, rheological analysis shows an increase in viscosity for P_2_W_17_-C_7_N due to higher elastic and viscous moduli, making it more suitable for coating applications and effectively preventing metal corrosion.

Some of the key properties of POM-IL, such as thermal stability, solubility, and viscosity, also significantly affect its application. For example, POM-ILs prepared from hydrophobic cations with alkyl chain length benefit from their application in corrosion inhibition, while POM-ILs containing hydroxyl or sulfonic acid groups can improve their hydrophilic properties. To reveal the relationship between structures and properties, five POM-ILs comprising Keggin-type POMs and tris(dihexylamino)cyclopropylene (TAC) cations are prepared, and their thermochemical and thermophysical properties are studied [100]. The results show that the melting points of the four-charged [PVMo_11_O_40_]^4−^ and [HPV_2_M_10_O_40_]^4−^ (M = Mo, W) are about 82 °C and those of PM with three charges are over 129 °C, which can be used for corrosion-resistance in the condition of high temperature.

### 8.2. Solar Cells

**Figure 14:**
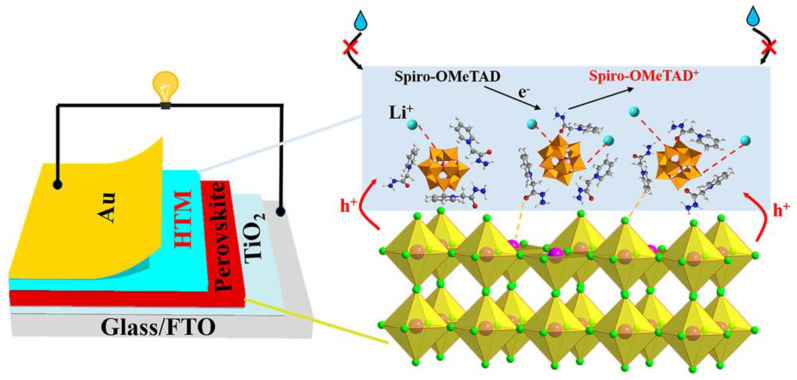
Schematic illustration for the structure of POM-IL modified PSC. Reproduced with permission from [102]. Copyright (2024) Elsevier.

Because of the reversible multi-electron redox capability, POM-ILs are excellent candidates for solar cells. For instance, four POM-ILs composed of inorganic clusters with [BMIM]^+^, 1-butylpyridinium, hexadecylpyridinium, and trihexyltetradecylphosphonium are applied in the dye-sensitized solar cells [101]. The efficacy of POM-ILs as photosensitizers is evaluated by their absorption on the photoanode (TiO_2_ film) for varying durations (15 min to 17 h), resulting in a 37-fold enhancement in the performance compared to commercially available compounds. POM-IL can also be applied in the field of perovskite solar cells (PSCs). Using phosphomolybdic acid and 1-(2-hydrazinyl-2-oxoethyl)pyridin-1-ium chloride as raw materials, a POM-IL is prepared and applied as an additive in PSC to improve its performance and stability [102]. The interaction of POM-IL with lithium bistrifluoromethane sulfonimide (Li-TFSI) inhibits the aggregation of Li-TFSI. The synergistic oxidation of POM-IL and Li-TFSI on 2,2′,7′-tetrakis[*N*,*N*-di(4-methoxyphenyl)amino]-9,9′-spirobifluorene (Spiro-OMeTAD) effectively improves the electrical properties of hole transport layer films and the photovoltaic properties of PSC (Figure 14), resulting in an excellent power conversion efficiency of 22.73%. Furthermore, the addition of POM@IL improves the humidity stability of the PSC, and after 1200 h of storage at high humidity (25 °C, 60% RH), the POM@IL modified device retains 81.2% of its initial power conversion efficiency.

## 9. Conclusions and Outlooks

POM-ILs are a combination of inorganic and organic fields, merging the characteristics of POMs and ILs, including good thermochemical stability, adjustable structure, excellent redox ability, etc. Various POM-ILs with diverse structures and properties have been explored based on different synthetic approaches. This paper highlights the recent applications of POM-ILs in catalysis, adsorption, batteries, antimicrobial, and anti-corrosion fields, with a particular emphasis on catalysis. Utilizing POMs, POM-ILs exhibit high oxidative activity in desulfurization reactions, alcohol transformation to aldehydes or ketones, olefin epoxidation, and lysis reactions. Due to the unique electronic structures of POMs, these catalytic reactions are efficiently carried out, showcasing distinct features when POM-ILs are used as catalysts. Additionally, the attractive structures of POM-ILs contribute to their exceptional performance in dye adsorption, lithium batteries, and antimicrobial and anti-corrosion applications.

Though POM-ILs have made rapid progress in recent decades, they still have significant development potential in the future. First, the discovery of new POM-IL structures remains a key focus. The structures of POM-ILs, as the foundation of their functions, require detailed study. In addition to delving deeply into existing structures, more attention should be given to the exploration of new architectures. Thanks to the rich variety of structures found in POMs and organic components, an increasing number of POM-ILs with innovative structures can be developed. Second, simplifying the synthesis process of POM-ILs is essential for their widespread application. As a type of functional material with versatile potential, POM-ILs are expected to be utilized not only in laboratories but also in industries. From a cost-saving perspective, a streamlined synthetic route for POM-ILs is crucial, playing a key role in their industrial applications. Moreover, the success of scaling up a reaction also depends on a simple and optimized synthetic strategy. Third, enhancing the catalytic ability of POM-ILs is an ongoing endeavor. As mentioned earlier, POM-ILs have been employed as catalysts for various reactions. However, compared to the diverse compositions and structures of POM-ILs, the range of catalytic reactions they have been used for is still limited. There is significant room for improvement in expanding the application of POM-ILs to a wider variety of reactions, which may exhibit unique reaction effects due to the distinctive features of POM-ILs. Additionally, improving current reactions catalyzed by POM-ILs is valuable, including reducing reaction times and temperatures, increasing conversion rates, and enhancing selectivity. Fourth, the thermodynamic properties of POM-IL are very important for its industrial application, but there are few studies on the thermodynamic properties of POM-IL, and the thermodynamic properties such as melting, glass transition, and decomposition temperatures, etc., should be studied in the future. Finally, the extensive functional expansion of POM-ILs is brimming with vitality. In addition to their role as catalysts, POM-ILs are anticipated to be utilized in a broader range of fields. The combination of POMs and ILs expands the structures of POM-ILs, as well as their properties. Through careful design and appropriate selection of the organic and inorganic parts, the prepared POM-ILs can express more interesting properties, which make it possible for the functional expansion of POM-ILs.

## Data Availability

Not applicable.

## Abbreviations

This section explains the abbreviations in the text.

DES	Deep eutectic solvents
PyPS	3-(Pyridine-1-ium-1-yl) propane-1-sulfonate
CoMo	Co(OH)_6_Mo_6_O_18_^3−^
MIM	1-Alkyl-3-methylimidazolium
GO	Graphene oxide
DBT	*p*-Dibenzothiophene
Vim	1-Vinyl-3-amylimidazolium
PM	H_3_PM_12_O_40_ (M = Mo, W)
DEDSA	Diethyldisulphoammonium
DBDSA	Dibutyldisulfoammonium
PET	Polyethylene terephthalate
BMIM	1-Butyl-3-methylimidazolium
Py	Pyridinium
TEAPs	1-(3-sulfonic group) triethylamine
MIMPs	1-methyl-3-(3-sulfopropyl)imidazolium
PMoV	H_5_PMo_10_V_2_O_40_
BSMIM	Butylsulfonate-3-methylimidazolium
PANI	Polyaniline
SAILEPs	Surface-active ionic liquid-encapsulated polyoxometalate
DDVAC	*N*,*N*-dimethyl-dodecyl-(4-vinylbenzyl) ammonium chloride
Co_4_PW	Na_10_[Co_4_(H_2_O)_2_(PW_9_O_34_)_2_]
NCA	LiNi_0.8_Co_0.15_Al_0.05_O_2_
RGO	Reduced GO
DOTMG	*N*,*N*,*N*′,*N*′-tetramethyl-*N*″,*N*″-dioctylguanidinum
MIC	Minimum inhibitory concentration
PMMA	Poly(methylmethacrylate)
SiW_11_	[α-SiW_11_O_39_]^8−^
P_44412_, P_44414_, and P_66614_	tribu-tyldodecyl, tributyltetradecyl, and trihexyltetradecyl
P_2_W_17_	K_10_[α_2-_P_2_W_17_O_61_]∙20H_2_O
P_2_V_3_W_15_	K_8_HP_2_W_15_V_3_O_62_·9H_2_O
TAC	tris(dihexylamino)cyclopropylene
PSCs	Perovskite solar cells
Li-TFSI	lithium bistrifluoromethane sulfonimide
Spiro-OMeTAD	2,2′,7′-tetrakis[N,N-di(4-methoxyphenyl)amino]-9,9′-spirobifluorene
